# Characteristics, Management, and Outcomes of Diabetes Subtypes in Patients With Cardiogenic Shock: A Nationwide Analysis

**DOI:** 10.1016/j.jacadv.2026.102766

**Published:** 2026-04-28

**Authors:** Ramy Sedhom, Nikitha Murthy, Michael Megaly, Anju Bhardwaj, Ofer Kobo, Marat Fudim, Mamas A. Mamas, Kevin Codorniz, Dmitry Abramov

**Affiliations:** a Department of Cardiology, Department of Medicine, Loma Linda University Health, Loma Linda, California, USA; b Department of Cardiology, Ascension St John Heart and Vascular Institute, Tulsa, Oklahoma, USA; c Department of Advanced Cardiopulmonary Therapies and Transplantation, The University of Texas–Houston, Houston, Texas, USA; d Department of Cardiology, Hillel Yaffe Medical Center, Hadera, Israel; e Keele Cardiovascular Research Group, Centre for Prognosis Research Keele University Stoke-on-Trent, United Kingdom; f Department of Medicine, Duke University Medical Center, Durham, North Carolina, USA; g Duke Clinical Research Institute, Durham, North Carolina, USA; h Keele Cardiovascular Research Group, Keele University, Keele, United Kingdom; i Division of Endocrinology, Loma Linda University Health, Loma Linda, California, USA

**Keywords:** cardiogenic shock, diabetes mellitus, type 1 diabetes, type 2 diabetes

## Abstract

**Background:**

There is a gap in current research looking at differences between diabetes subtypes in the setting of cardiogenic shock (CS).

**Objectives:**

The authors sought to evaluate differences in characteristics, management, and outcomes between patients with no diabetes mellitus (DM), type 1 DM (T1DM), and type 2 DM (T2DM) admitted with CS.

**Methods:**

The National Readmissions Database was utilized to identify patients between 2016 and 2021 with any discharge diagnosis of CS. The cohort was divided into 3 groups: CS without DM, CS with T1DM, and CS with T2DM. The primary outcomes were all-cause in-hospital mortality and 30-day readmissions.

**Results:**

A total of 1,073,340 patients hospitalized with CS were identified during the study period, of which 644,768 (60.0%) had no DM, 10,423 (1.0%) had T1DM, and 418,149 (39.0%) had T2DM. Those with T1DM were more likely to present with CS due to acute myocardial infarction and undergo invasive evaluation with left heart catheterization compared to those with T2DM and no DM. In-hospital mortality was higher among those with T1DM (adjusted OR: 1.16; 95% CI: 1.09-1.24) and T2DM (adjusted OR: 1.08; 95% CI: 1.07-1.10) compared to those without DM, and both groups were more likely to be readmitted within 30 days. Among patients with CS from acute myocardial infarction, in-hospital mortality was similar between patients with T1DM and no DM, but higher in those with T2DM.

**Conclusions:**

Patients with DM, particularly T1DM, who present with CS represent a high-risk cohort with greater in-hospital mortality and higher readmission rates compared to patients without DM.

Cardiogenic shock (CS) is commonly encountered in the cardiac intensive care unit and is associated with significant morbidity and mortality. CS is commonly caused by acute myocardial infarction (AMI); however, other causes include end-stage cardiomyopathy, myocarditis, valvular insufficiency, and other causes.^1^^,^^2^ The epidemiology of CS has changed over time, particularly with a greater prevalence of non-AMI CS.^3^^,^^4^ The risk of mortality in patients who develop CS depends on various factors, including shock severity, magnitude of end-organ dysfunction, and specific patient characteristics.^5^ Diabetes mellitus (DM) is a common comorbidity in patients presenting with CS and may increase the likelihood of developing CS by 2 to 3 times compared to those without DM.^6^^,^^7^ While several cohorts of patients with DM and CS have been described,^7^^,^^8^ there are remaining gaps in the literature regarding the management and outcomes of patients with DM and CS in the United States in the current era. Particularly, there are limited data on the in-hospital management and outcomes of patients with CS among both AMI and non-AMI cohorts, and cohorts further characterized by type 1 DM (T1DM) vs type 2 DM (T2DM). As such, we utilized the National Readmissions Database to further evaluate the management and outcomes including mortality and readmissions of patients with both AMI and non-AMI CS, particularly subcharacterized by DM type.

## Study design and methods

### Data source

The National Readmissions Database was utilized to extract data from this study between the years of 2016 and 2021. Details regarding the National Readmissions Database have been published elsewhere.^9^^,^^10^ Baseline characteristics, outcomes, and procedures were identified using the International Classification of Diseases (ICD)-Tenth Revision-Clinical Modification and ICD-Tenth Revision-Procedure Coding System codes (Supplemental Table 1). The National Readmissions Database is a publicly available database with deidentified hospitalization records; as such, this study was exempt from Institutional Review Board approval.

### Study population

All patients aged 18 years or older with a discharge diagnosis of CS from 2016 to 2021 were included. Of this cohort, we then identified patients who had T1DM and T2DM (ICD codes provided in Supplemental Table 1). ICD codes are commonly utilized for population-based research and have been found to have acceptable sensitivity and specificity for classifying conditions such as DM.^9^^,^^10^ The study cohort was divided into 3 groups: CS without DM, CS with T1DM, and CS with T2DM. For the readmission analysis, we excluded those admitted in December (since the National Readmissions Database does not cross the calendar year) and those who died during the index admission.^11^^,^^12^

### Outcomes

The primary outcomes were the differences in all-cause in-hospital mortality and urgent 30-day readmission rates between the study groups. Secondary outcomes included the differences in the incidence of cardiac arrest, ventricular tachycardia, ischemic stroke, intracerebral hemorrhage, major extracranial hemorrhage, acute kidney injury, and acute limb ischemia. These outcomes were assessed by secondary discharge diagnosis codes from the initial hospitalization. We also examined the differences in length and cost of stay. Length of stay was calculated by subtracting the admission date from the discharge date.^13^ Hospital costs were calculated using hospital-specific cost-to-charge ratios provided by the healthcare cost and utilization project.^14^

### Statistical analysis

Analyses were conducted using multilevel complex analysis to account for hospital clustering, weights, and stratification and as such, complying by Healthcare Cost and Utilization Project regulations. Continuous variables were summarized as medians and 25–75th percentiles (Q1-Q3) and compared with the Kruskal-Wallis test. Categorical variables are displayed as numbers and percentages and compared with Pearson’s chi-square or Fisher exact test. A 2-sided *P* value <0.05 was considered statistically significant.

Multivariable logistic regression analyses were used to account for the differences in the baseline patient and hospital characteristics, with results presented as the adjusted OR (aOR) with 95% CI. The following variables were included in the multivariable analyses: age (10-year increments), female sex, transfer status, morbid obesity, smoking, hypertension, atrial fibrillation, coronary artery disease, peripheral arterial disease, cerebrovascular accident, ventricular assist device, chronic kidney disease, lung disease, liver disease, novel-coronavirus infection, anemia, MI, hospital size and teaching status, and Medicare insurance status. Linear regression was used for trend analysis. Additionally, we performed subgroup analysis for patients with AMI cardiogenic shock (AMI-CS). Statistical analyses were performed using IBM SPSS Statistics for Windows, Version 29.0 (IBM Corp).

## Results

### Prevalence of DM and CS

**Figure 1:**
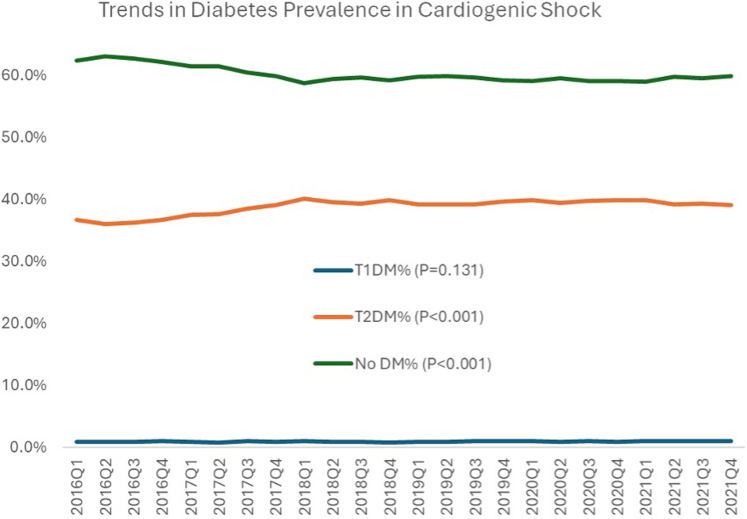
**Trends in the Prevalence of Diabetes Mellitus in Cardiogenic Shock** DM = diabetes mellitus; T1DM = type 1 diabetes mellitus; T2DM = type 2 diabetes mellitus.

From 2016 to 2021, 1,073,340 weighted hospitalizations for patients who developed CS were identified. Of these, a total of 428,572 hospitalizations had a documented diagnosis of DM (39.9%). Of the total population, 10,423 (1.0%) had T1DM and 418,149 (39.0%) had T2DM. The trends in the prevalence of DM among CS admissions are shown in Figure 1, with a rise in the prevalence of T2DM during the study period. Of the total CS hospitalizations, 358,401 hospitalizations were for AMI-CS, of which 204,303 (57.0%) had no DM, 2,075 (0.6%) had T1DM, and 149,809 (41.8%) had T2DM.

### Characteristics of patients with T1DM, T2DM, and no DM who developed CS

The characteristics of patients with T1DM, T2DM, and no DM hospitalized with CS are presented in Table 1. Patients with T1DM were younger, more likely to be female, and had a higher prevalence of certain comorbidities including chronic kidney disease, end-stage kidney disease, and anemia compared to patients with T2DM and those without DM. Patients with T2DM hospitalized with CS were older and had a higher prevalence of certain comorbidities including morbid obesity, smoking, hypertension, atrial fibrillation, coronary artery disease, cerebrovascular accident, chronic lung disease, and COVID-19. Patients with T1DM were more likely to develop CS due to AMI and therefore were more likely to undergo invasive evaluation with left heart catheterization (34.9%) compared to patients with T2DM (32.0%) and no DM (28.9%). The utilization of right heart catheterization was 16.5% in patients with T1DM, 17.0% in patients with T2DM, and 15.9% in patients without DM. The utilization of mechanical ventilation, intra-aortic balloon pump, Impella, and extracorporeal membrane oxygenation was also more common among those with T1DM (Table 2).

**Table 1:** Characteristics of Patients With T1DM, T2DM, and No DM Hospitalized With CS

	CS Without DM (n = 644,768)	CS With TIDM (n = 10,423)	CS With T2DM (n = 418,149)	*P* Value
Age (y)	67 (57-77)	59 (47-69)	69 (60-77)	<0.001
Female	242,713 (37.6%)	4,870 (46.7%)	154,504 (36.9%)	<0.001
Transferred from other hospitals	61,960 (9.6%)	1,096 (10.5%)	51,171 (12.2%)	<0.001
Comorbidities				
Morbid obesity	39,264 (6.1%)	664 (6.4%)	57,309 (13.7%)	<0.001
Smokers	131,563 (20.4%)	1,613 (15.5%)	91,022 (21.8%)	<0.001
Hypertension	446,210 (69.2%)	8,313 (79.8%)	368,156 (88.0%)	<0.001
Atrial fibrillation	283,903 (44.0%)	2,863 (27.5%)	192,162 (46.0%)	<0.001
CAD	317,331 (49.2%)	6,349 (60.9%)	271,201 (64.9%)	<0.001
PAD	55,925 (8.7%)	888 (8.5%)	33,245 (8.0%)	<0.001
CVA	54,713 (8.5%)	983 (9.4%)	47,442 (11.3%)	<0.001
LVAD	6,005 (0.9%)	102 (1.0%)	3,941 (0.9%)	0.89
Chronic lung disease	169,340 (26.3%)	1,851 (17.8%)	116,463 (27.9%)	<0.001
CKD	196,650 (30.5%)	5,655 (54.3%)	214,791 (51.4%)	<0.001
ESKD	33,661 (5.2%)	2,352 (22.6%)	50,876 (12.2%)	<0.001
Liver disease	19,956 (3.1%)	202 (1.9%)	11,456 (2.7%)	<0.001
Anemia	264,300 (41.0%)	4,421 (42.4%)	171,127 (40.9%)	0.22
COVID-19	13,830 (2.1%)	224 (2.1%)	11,490 (2.7%)	<0.001
Triggering event				
AMI	204,303 (31.7%)	4,289 (41.2%)	149,809 (35.8%)	<0.001
Anterior STEMI	44,836 (7.0%)	628 (6.0%)	24,871 (5.9%)	<0.001
Myocarditis	4,657 (0.7%)	46 (0.4%)	1,333 (0.3%)	<0.001
Hospital and payer				
Teaching hospital	524,820 (81.4%)	8,630 (82.8%)	338,897 (81.0%)	0.014
Large hospital	433,362 (67.2%)	7,050 (67.6%)	275,734 (65.9%)	<0.001
Medicare	384,962 (59.7%)	6,114 (58.7%)	283,726 (67.9%)	<0.001

Values are median (25–75th percentiles) or n (%) and compared using the chi-squared or Kruskal-Wallis test.

AMI = acute myocardial infarction; CAD = coronary artery disease; CKD = chronic kidney disease; CS = cardiogenic shock; CVA = cerebrovascular accident; DM = diabetes mellitus; ESKD = end-stage kidney disease; LVAD = left ventricular assist device; PAD = peripheral artery disease; STEMI = ST-segment elevation myocardial infarction; T1DM = type 1 diabetes mellitus; T2DM = type 2 diabetes mellitus.

**Table 2:** Utilization of Mechanical Ventilation, Invasive Testing, MCS, and Revascularization in all Causes of CS Among Those With T1DM, T2DM, and No DM

	CS Without DM (n = 644,768)	CS With T1DM (n = 10,423)	CS With T2DM (n = 418,149)
Mechanical ventilation	299,956 (46.5%)	5,129 (49.2%)	193,328 (46.2%)
RHC	102,543 (15.9%)	1,720 (16.5%)	71,012 (17.0%)
LHC	186,284 (28.9%)	3,637 (34.9%)	133,785 (32.0%)
Any MCS	126,760 (19.7%)	2,353 (22.6%)	85,586 (20.5%)
IABP	84,795 (13.2%)	1,633 (15.7%)	60,680 (14.5%)
Impella	38,100 (5.9%)	718 (6.9%)	26,484 (6.3%)
ECMO	19,547 (3.0%)	320 (3.1%)	7,914 (1.9%)
Any revascularization	151,091 (23.4%)	3,179 (30.5%)	114,721 (27.4%)
PCI	95,229 (14.8%)	1,742 (16.7%)	64.774 (15.5%)
CABG	59,069 (9.2%)	1,495 (14.3%)	52,406 (12.5%)

Values are n (%). Event rates were not available so death could not be considered as a competing risk; thus, event rates may be underestimated.

CABG = coronary artery bypass grafting; ECMO = extracorporeal membrane oxygenation; IABP = intra-aortic balloon pump; LHC = left heart catheterization; MCS = mechanical circulatory support; PCI = percutaneous coronary intervention; RHC = right heart catheterization; other abbreviations as in Table 1.

The management of patients with DM and CS among only the cohort who presented with AMI-CS is shown in Supplemental Table 2. Utilization of right heart catheterization and left heart catheterization was highest in patients with T1DM while utilization of any mechanical circulatory support (MCS) was clinically similar between the groups. Patients with T1DM and T2DM were more likely to undergo revascularization with coronary artery bypass grafting and less likely to undergo revascularization with percutaneous coronary intervention compared to patients without DM.

### Primary outcomes

**Figure 2:**
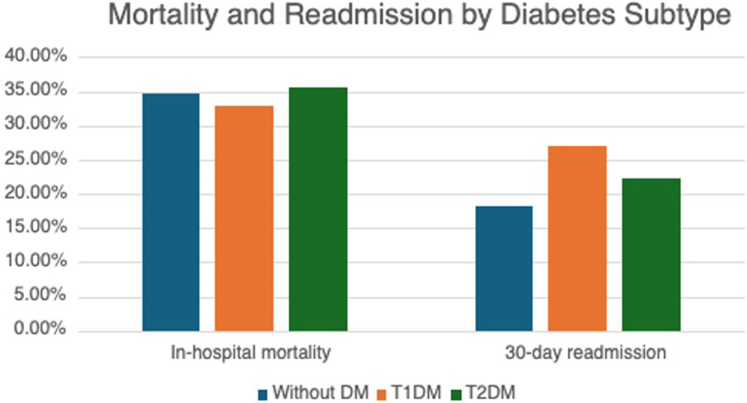
**Unadjusted Rates of In-hospital Mortality and 30-Day Readmission in Patients With Type 1 Diabetes Mellitus, Type 2 Diabetes Mellitus, and No Diabetes Mellitus** Abbreviations as in Figure 1.

The unadjusted outcomes among patients with T1DM, T2DM, and without DM are shown in Table 3. Unadjusted in-hospital mortality was 34.7% in CS patients without DM, 32.8% in those with T1DM, and 35.6% in those with T2DM (*P* < 0.001). Patients with T1DM had the greatest hospital costs and were more likely to be readmitted at 30 days (26.9% vs 22.2% for patients with T2DM and 18.3% for patients without DM) (Figure 2).

**Table 3:** Unadjusted Outcomes of CS in Patients Without DM, T1DM, and T2DM

	CS Without DM (n = 644,768)	CS With Type I DM (n = 10,423)	CS With Type II DM (n = 418,149)
In-hospital mortality	223,725 (34.7%)	3,417 (32.8%)	148,840 (35.6%)
Cardiac arrest	133,618 (20.7%)	2,296 (22.0%)	79,922 (19.1%)
VT	129,457 (20.1%)	1,885 (18.1%)	82,990 (19.8%)
Discharge to a facility	140,594 (21.8%)	2,491 (23.9%)	99,398 (23.8%)
Ischemic stroke	18,021 (2.8%)	298 (2.9%)	10,864 (2.6%)
ICH	11,155 (1.7%)	95 (0.9%)	4,929 (1.2%)
AKI	400,934 (62.2%)	6,608 (63.4%)	275,963 (66.0%)
Acute limb ischemia	8,351 (1.3%)	144 (1.4%)	4,103 (1.0%)
Major extracranial bleeding	148,942 (23.1%)	2,342 (22.5%)	92,807 (22.2%)
Blood transfusion	69,547 (10.8%)	1,352 (13.0%)	50,419 (12.1%)
LOS (d)	8 (4-16)	9 (4-18)	9 (4-16)
Cost (dollars)	38,374 (19,006-75,468)	44,216 (22,750-79,795)	39,146 (19,735-73,708)
Urgent 30-d readmission^a^	70,031/382,284 (18.3%)	1,730/6,440 (26.9%)	54,277/244,572 (22.2%)
Time to readmission (d)^b^	10 (4-18)	9 (4-17)	10 (4-18)

Values are median (25–75th percentiles) or n (%). Event rates were not available so death could not be considered as a competing risk; thus, event rates may be underestimated.

AKI = acute kidney injury; ICH = intracerebral hemorrhage; LOS = length of stay; VT = ventricular tachycardia; other abbreviations as in Table 1.

a After excluding those who died during index hospitalization and those who were admitted in December of each calendar year.

b Refers only to patients who were readmitted within 30 days.

Multivariable analyses for mortality and other key outcomes are shown in Table 4. In-hospital mortality was higher among both those with T1DM (aOR: 1.16; 95% CI: 1.09-1.24) and T2DM (aOR: 1.08; 95% CI: 1.07-1.10) compared to those without DM. Among patients with DM and CS, patients with T1DM had higher odds of in-hospital mortality compared to patients with T2DM (aOR: 1.09; 95% CI: 1.02-1.16). Thirty-day readmission was more likely to occur among patients with T1DM (aOR: 1.45; 95% CI: 1.33-1.58) and T2DM (aOR: 1.19; 95% CI: 1.16-1.21) compared to patients without DM.

**Table 4:** Adjusted Odds Ratio of In-Hospital Outcomes in Those With T1DM and T2DM (Reference Group is CS Without DM)

	T1DM	T2DM
In-hospital mortality	1.16 (1.09-1.24)	1.08 (1.07-1.10)
Urgent 30-d readmission	1.45 (1.33-1.58)	1.19 (1.16-1.21)

Values are OR (95% CI). Data do not account for competing risks of outcomes.

Abbreviations as in Table 1.

In outcome analyses limited to patients with AMI-CS, in-hospital mortality was 34.6% in patients without DM, 29.9% in patients with T1DM, and 36.5% in patients with T2DM. Multivariable analyses for outcomes limited to CS-AMI are shown in Supplemental Table 3. Compared to patients without DM, in-hospital mortality was similar in patients with T1DM but was higher in patients with T2DM. Urgent 30-day readmissions were significantly higher in patients with T1DM compared to patients with T2DM and to patients without DM.

## Discussion

This analysis on the in-hospital management and outcomes of CS among patients with T1DM and T2DM compared to patients without DM demonstrates several important findings (Central Illustration). Patients with T1DM were younger, more likely to be female, and had a higher prevalence of chronic kidney disease, while patients with T2DM had a higher prevalence of obesity, hypertension, coronary artery disease, and atrial fibrillation. Patients with T1DM were most likely to present with AMI leading to CS and were more likely to undergo invasive evaluation and revascularization during their CS hospitalization compared to patients with T2DM and those without DM. In multivariable analyses, patients with both types of DM had higher mortality and 30-day readmissions compared to patients without DM. Among patients with CS limited to AMI-CS, 30-day readmission was highest in patients with T1DM, while in-hospital mortality was higher in patients with T2DM but similar between patients with T1DM and those without DM. These results have important clinical implications for understanding the care and outcomes of patients with T1DM and T2DM who are hospitalized with CS.

**Central Illustration:**
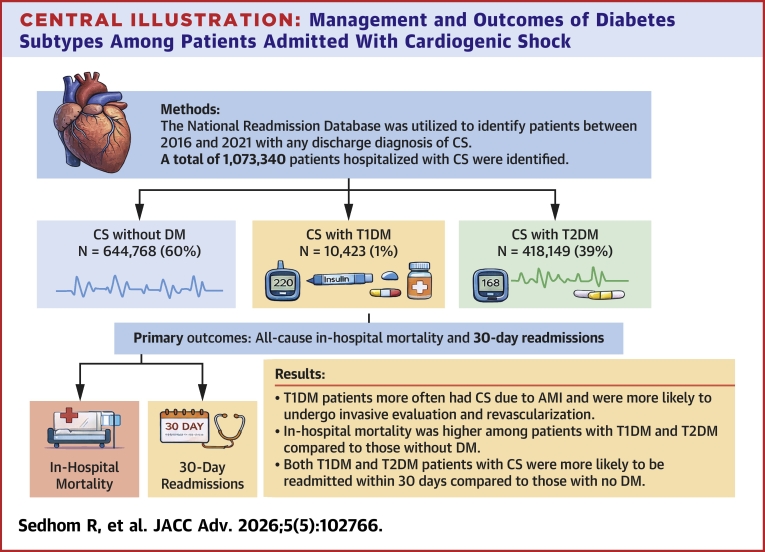
**Management and Outcomes of Diabetes Subtypes Among Patients Admitted With Cardiogenic Shock** CS = cardiogenic shock; other abbreviations as in Figure 1.

Existing literature on CS in patients with DM has focused primarily on AMI-CS, in part because DM is a major risk factor for AMI among both men and women.^15^^,^^16^ Patients with DM have a 2 to 3 times heightened risk of developing CS in the setting of AMI compared to those without DM.^7^^,^^8^^,^^17^, ^18^, ^19^ This exacerbated risk may be related to microvascular and macrovascular changes, larger infarct size, increased reperfusion injury, increased predisposition to arrhythmias, lower myocardial functional reserve, and potentially greater burden or comorbidity severity in those with DM.^20^, ^21^, ^22^, ^23^, ^24^, ^25^, ^26^ Similar pathophysiology differences between patients with and without DM, including differences that lead to the development of diabetic cardiomyopathy,^27^ may also increase the risks of non-AMI CS. Although patients with AMI-CS are more likely to have DM than patients who present with other etiologies of CS, DM is overall a common comorbidity in all patients with CS^28^ and prior data are in line with ours which suggest that approximately 40.0% of patients with CS have underlying DM. However, there are gaps in the literature on the management and outcomes of CS in patients with DM in the current era and for patients presenting with AMI-CS and other etiologies of CS. Additionally, only limited data are available on the differences in evaluation and management of CS between patients characterized by T1DM and T2DM.

Our results identify important differences in the presentation and management of patients with CS based on not only the presence of DM but on the differences between patients with T1DM and T2DM. Patients with T1DM were younger and had different patterns of underlying comorbidities compared to patients with T2DM and those without DM. Patients with T1DM presenting with CS were more likely to have chronic kidney disease and end-stage kidney disease at the time of presentation and to present with AMI-CS compared to patients without DM and those with T2DM. This may be due to pathophysiological and treatment differences between patients with T1DM and T2DM, where patients with T1DM demonstrate greater risks for cardiovascular disease compared to not only patients without DM but also patients with T2DM.^29^ Differences in patient characteristics and presentation were also associated with differences in the management of CS, with higher utilization of invasive coronary evaluation and MCS in those with T1DM. Higher rates of invasive coronary evaluation and utilization of MCS, including intra-aortic balloon pump, extracorporeal membrane oxygenation, and Impella, in patients with T1DM may be due to several factors, including younger age at presentation, higher likelihood of AMI which was perhaps considered to be a more reversible cause of CS and where management often occurs in the catheterization lab where MCS is performed, as well as a lower prevalence of certain comorbidities such as chronic lung disease and liver disease which may otherwise be considered contraindications for complex mechanical support. Among the cohort presenting with AMI CS, invasive evaluation was highest in patients with T1DM compared to patients without DM and those with T2DM, and there were differences in revascularization where patients with T1DM and T2DM were more likely to undergo revascularization with coronary artery bypass grafting and less likely to undergo percutaneous coronary intervention compared to patients without DM. Although the effects of revascularization on CS outcomes could not be evaluated in our analysis, prior data demonstrate that patients with DM have similar benefit from revascularization compared to those without DM in the presence of AMI-CS.^30^ This suggests that invasive evaluation and revascularization are important treatment options in the setting of CS irrespective of DM status.

We also examine variations in in-hospital outcomes for patients with CS, comparing those with DM to those without. Our results expand upon prior data which generally demonstrated an increased mortality in CS patients with DM. However, those data were primarily limited to AMI-CS. Additionally, our results highlight differences in outcomes between T1DM and T2DM, demonstrating that among all patients with CS, patients with T1DM had higher mortality than patients with T2DM. However, among patients only with CS due to AMI, patients with T2DM had higher mortality while mortality among patients with T1DM and those without DM were similar. These results differ from a prior older study of patients with AMI-CS which did not demonstrate significant outcome differences between patients with T1DM and T2DM,^8^ with differences potentially related to greater severity of comorbidities associated with DM in the current era.

These data have important clinical implications as they highlight the current evaluation and management of the large number of patients with DM who present with CS. Identifying those at elevated risk of mortality and readmission from time of admission can help increase awareness and encourage early intervention and medication optimization in those with T1DM and T2DM, which is important as the prevalence of DM has increased over time. Particularly, patients with T1DM who develop CS might be at an increased risk of in-patient complications and readmission and require early recognition. Further studies will be needed on ways to optimize care in this high-risk cohort, including on better risk stratification for which patients may derive benefit from invasive management with MCS. For example, the role of pre-existing DM control and the potential for subclinical or undiagnosed DM complications which may affect acute clinical care and outcomes will require further evaluation. Decision-making on acute intervention in patients with DM and CS may also take into account potential candidacy for advanced therapy options, including heart transplantation and durable ventricular assist device. The prevalence of DM among patients undergoing advanced therapies in the United States has increased over time, although long-term outcomes may be worse compared to the non-DM cohorts which may affect decision-making for CS care.^31^^,^^32^ In-hospital management should also focus on hyperglycemia, which has been associated with higher in-hospital mortality with CS.^33^ Additional studies in the current era are also needed on the role of diabetic cardiomyopathy in all-cause and AMI CS.

### Study Limitations

Our study has limitations. The National Readmissions Database data are reliant on ICD coding; as such, inaccurate coding could play a significant role correctly identifying those with DM or other comorbidities. The etiology of CS, other than from AMI, was not further characterized. We were unable to obtain data regarding duration of DM, control of DM, and medication utilization, and data on the severity of CS were not available. The reasons for decision-making, such as revascularization, are not available in the National Readmissions Database. Specific causes of mortality are not available. The temporal relationship between inpatient outcomes and postdischarge mortality data are not available in the National Readmissions Database, which affects the ability to perform competing risk analyses. The National Readmissions Database does not report data on mortality after hospital discharge; therefore, readmission rates must be interpreted in the context that some patients may have died prior to readmission. Race and ethnicity data are not available in the National Readmissions Database.

## Conclusions

We highlight differences in the in-hospital management and outcomes of patients with all-cause CS and CS due to AMI among patients with T1DM, patients with T2DM, and patients without DM. Patients with T1DM who present with CS represent a high-risk cohort, with greater in-hospital mortality and higher readmission rates compared to patients with T2DM and patients without DM. Additional research will be needed to optimize the outcomes of the patients with DM who present with CS.

## Funding support and author disclosures

Dr Fudim has received personal fees from Alleviant, Ajax, Alio Health, Alleviant, Artha, Audicor, Axon Therapies, 10.13039/100004326Bayer, Bodyguide, Bodyport, 10.13039/100008497Boston Scientific, Broadview, Cadence, Cardioflow, Cardionomics, Coridea, CVRx, Daxor, Deerfield Catalyst, 10.13039/100006520Edwards LifeSciences, Echosens, EKO, Feldschuh Foundation, Fire1, FutureCardia, Galvani, Gradient, Hatteras, HemodynamiQ, ImpulseDynamics, Intershunt, 10.13039/100004374Medtronic, 10.13039/100004334Merck, NIMedical, NovoNordisk, NucleusRx, NXT Biomedical, Orchestra, Pharmacosmos, PreHealth, Presidio, Procyreon, ReCor, Rockley, SCPharma, Shifamed, Splendo, Summacor, SyMap, Verily, Vironix, Viscardia, and Zoll; and has received grants from the 10.13039/100000002National Institutes of Health, Doris Duke. Dr Codorniz has received fees from Medtronic and Mannkind. Dr Abramov has received speaker fees from AstraZeneca and 10.13039/100004326Bayer. All other authors have reported that they have no relationships relevant to the contents of this paper to disclose.

## References

[1] Samsky M.D., Morrow D.A., Proudfoot A.G., Hochman J.S., Thiele H., Rao S.V. (2021). Cardiogenic shock after acute myocardial infarction: a review. JAMA.

[2] Topalian S., Ginsberg F., Parrillo J.E. (2008). Cardiogenic shock. Crit Care Med.

[3] Sinha S.S., Rosner C.M., Tehrani B.N. (2022). Cardiogenic shock from heart failure versus acute myocardial infarction: clinical characteristics, hospital course, and 1-year outcomes. Circ Heart Fail.

[4] Jentzer J.C., Ahmed A.M., Vallabhajosyula S. (2021). Shock in the cardiac intensive care unit: changes in epidemiology and prognosis over time. Am Heart J.

[5] Vahdatpour C., Collins D., Goldberg S. (2019). Cardiogenic shock. J Am Heart Assoc.

[6] Lindholm M.G., Boesgaard S., Torp-Pedersen C., Køber L. (2005). Diabetes mellitus and cardiogenic shock in acute myocardial infarction. Eur J Heart Fail.

[7] Luo C., Chen F., Liu L., Ge Z., Feng C., Chen Y. (2022). Impact of diabetes on outcomes of cardiogenic shock: a systematic review and meta-analysis. Diab Vasc Dis Res.

[8] Echouffo-Tcheugui J.B., Kolte D., Khera S. (2018). Diabetes mellitus and cardiogenic shock complicating acute myocardial infarction. Am J Med.

[9] Chi G.C., Li X., Tartof S.Y., Slezak J.M., Koebnick C., Lawrence J.M. (2019). Validity of ICD-10-CM codes for determination of diabetes type for persons with youth-onset type 1 and type 2 diabetes. BMJ Open Diabetes Res Care.

[10] Khokhar B., Jette N., Metcalfe A. (2016). Systematic review of validated case definitions for diabetes in ICD-9-coded and ICD-10-coded data in adult populations. BMJ Open.

[11] NRD overview. https://hcup-us.ahrq.gov/nrdoverview.jsp.

[12] Sedhom R., Elbadawi A., Megaly M. (2022). Hospital procedural volume and outcomes with catheter-directed intervention for pulmonary embolism: a nationwide analysis. Eur Heart J Acute Cardiovasc Care.

[13] Healthcare cost and utilization project (HCUP) NRD notes. https://hcup-us.ahrq.gov/db/vars/los/nrdnote.jsp.

[14] Cost H., Project U. (Published online 2010).

[15] Millett E.R.C., Peters S.A.E., Woodward M. (2018). Sex differences in risk factors for myocardial infarction: cohort study of UK biobank participants. BMJ.

[16] Schmitt V.H., Hobohm L., Hahad O. (2025). Impact of type 1 diabetes mellitus on mortality rate and outcome of hospitalized patients with myocardial infarction. Diabetes & Diabetes Metab Syndr.

[17] De Ferranti S.D., De Boer I.H., Fonseca V. (2014). Type 1 diabetes mellitus and cardiovascular disease: a scientific statement from the American Heart Association and American Diabetes Association. Circulation.

[18] Tedesco J.V., Wright R.S., Williams B.A. (2003). Effect of diabetes on the mortality risk of cardiogenic shock in a community-based population. Mayo Clin Proc.

[19] Dauriz M., Morici N., Gonzini L. (2020). Fifteen-year trends of cardiogenic shock and mortality in patients with diabetes and acute coronary syndromes. Am J Med.

[20] Gatuz M.V., Abu-Fanne R., Abramov D., Mamas M.A., Roguin A., Kobo O. (2024). Diabetes and its impact on cardiogenic shock outcomes in acute myocardial infarction with polyvascular disease: a comparative analysis. Biomedicines.

[21] Gregg E.W., Sattar N., Ali M.K. (2016). The changing face of diabetes complications. Lancet Diabetes Endocrinol.

[22] Alegria J.R., Miller T.D., Gibbons R.J., Yi Q.L., Yusuf S. (2007). Infarct size, ejection fraction, and mortality in diabetic patients with acute myocardial infarction treated with thrombolytic therapy. Am Heart J.

[23] Bakth S., Arena J., Lee W. (1986). Arrhythmia susceptibility and myocardial composition in diabetes. Influence of physical conditioning. J Clin Invest.

[24] Movahed M.R., Hashemzadeh M., Jamal M. (2007). Increased prevalence of ventricular fibrillation in patients with type 2 diabetes mellitus. Heart Vessels.

[25] Ha J.W., Lee H.C., Kang E.S. (2007). Abnormal left ventricular longitudinal functional reserve in patients with diabetes mellitus: implication for detecting subclinical myocardial dysfunction using exercise tissue Doppler echocardiography. Heart.

[26] Lejay A., Fang F., John R. (2016). Ischemia reperfusion injury, ischemic conditioning and diabetes mellitus. J Mol Cell Cardiol.

[27] Shou Y., Li X., Fang Q. (2024). Progress in the treatment of diabetic cardiomyopathy, a systematic review. Pharmacol Res Perspect.

[28] Schrage B., Weimann J., Dabboura S. (2020). Patient characteristics, treatment and outcome in non-ischemic vs. ischemic cardiogenic shock. J Clin Med.

[29] Larsson S.C., Wallin A., Håkansson N., Stackelberg O., Bäck M., Wolk A. (2018). Type 1 and type 2 diabetes mellitus and incidence of seven cardiovascular diseases. Int J Cardiol.

[30] Shindler D.M., Palmeri S.T., Antonelli T.A. (2000). Diabetes mellitus in cardiogenic shock complicating acute myocardial infarction: a report from the SHOCK Trial Registry. J Am Coll Cardiol.

[31] Crugnola W., Cinquina A., Mattimore D. (2024). Impact of diabetes mellitus on outcomes in patients with left ventricular assist devices. Biomedicines.

[32] Stepanova M., Kumar A., Brandt P. (2023). Impact of type 2 diabetes on the outcomes of solid organ transplantations in the US: data from a national registry. Diabetes Care.

[33] Kataja A., Tarvasmäki T., Lassus J. (2017). The association of admission blood glucose level with the clinical picture and prognosis in cardiogenic shock – results from the cardshock study. Int J Cardiol.

