# Supplementary material for: Characteristics, Management, and Outcomes of Diabetes Subtypes in Patients With Cardiogenic Shock: A Nationwide Analysis
Source: JACC Adv. 2026 Apr 28;5(5):102766. doi: 10.1016/j.jacadv.2026.102766 (PMC13140053; doi:10.1016/j.jacadv.2026.102766)
Supplement: Supplemental Material [file mmc1.docx]

**Supplemental Table 1.** International Classification of Diseases, Tenth Revision codes used in the study.

| **Diagnosis** | **ICD-10 CM code** |
| --- | --- |
| **Type I DM** | Without complications  E10.10, E10.11, E10.19, E10.9, E10.8  Renal complications  E10.21, E10.22, E10.29, E13.291, E13.292, E13.293, E13.299  Ophthalmic complications  E10.31, E10.32, E10.33, E10.39, E13.311, E13.312, E13.313, E13.319  Neurologic complications  E10.41, E10.42, E10.43, E10.49, E13.541, E13.542, E13.543, E13.549  Circulatory complications  E10.51, E10.52, E10.53, E10.59, E13.551, E13.552, E13.553, E13.559  Other specified complications  E10.61, E10.62, E10.63, E10.69, E13.591, E13.592, E13.593, E13.599  Multiple complications  E10.7x series  Unspecified/other  E10.4x, E13.211–E13.219 (as applicable) |
| **Type II DM** | Without complications  E11.00, E11.01, E11.10, E11.11, E11.29, E11.9, E11.9  Renal complications  E11.21, E11.22, E11.29, E11.391, E11.392, E11.393, E11.399  Ophthalmic complications  E11.31, E11.32, E11.33, E11.39, E11.411, E11.412, E11.413, E11.419  Neurologic complications  E11.41, E11.42, E11.43, E11.49, E11.51, E11.52, E11.53, E11.59  Circulatory complications  E11.61, E11.62, E11.63, E11.69, E11.7x series  Other specified complications  E11.6, E11.8, E11.9, E11.10–E11.19  Unspecified/other  E11.9, E11.00–E11.99 (as applicable) |
| **Cardiogenic shock** | R570, T8111XA, T8111XD, T8111XS |
| **Myocardial infarction** | |
| *STEMI* | I210, I2101, I2102, I513, I2109, I211, I2111, I2119, I212 |
| *NSTEMI* | I214 |
| **Intracerebral Hemorrhage** | Non-traumatic subarachnoid hemorrhage: I60 (I60.0 – I60.9)  Non-traumatic intracerebral hemorrhage: I61 (I61.0 – I61.9)  Other and unspecified nontraumatic intracranial hemorrhage: I62 (I62.0 – I62.9)  Sequelae of intracranial hemorrhage: I69.0-I69.2  Epidural hemorrhage: S06.4(x) |
| **Gastrointestinal bleeding** | Gastric ulcer with bleeding: K25.0, K25.2, K25.4, K25.6 Duodenal ulcer with bleeding: K26.0, K26.2, K26.4, K26.6  Peptic ulcer with bleeding: K27.0, K27.2, K27.4, K27.6  Gastrojejunal ulcer with bleeding: K28.0, K28.2, K28.4, K28.6  Acute gastritis with bleeding: K29.01  Esophageal varices with bleeding: I85.01  Ulcer of esophagus with bleeding: K22.11  Hematemesis: K92.0  Melena: K92.1  Angiodysplasia of colon with hemorrhage: K55.21  Hemorrhage of anus and rectum: K62.5  Gastrointestinal hemorrhage, unspecified: K92.2 |
| **Hemoperitoneum** | K66.1 |
| **Thoracic and respiratory bleeding** | Hemothorax: J94.2  Hemorrhage from respiratory passages: R04  Epistaxis: R04.0  Hemorrhage from throat: R04.1  Hemoptysis: R04.2  Hemorrhage from other sites in respiratory passages: R04.8  Hemorrhage from other sites in respiratory passages: R04.89  Hemorrhage from respiratory passages, unspecified: R04.9 |
| **Hematuria** | Recurrent and persistent hematuria N02.(x)  Hematuria: R31  Gross hematuria: R31.0  Hematuria, unspecified: R31.9 |
| **Gynecological bleeding** | Abnormal uterine and vaginal bleeding: N93.8, N93.9  Postmenopausal bleeding: N95.0  Hematosalpinx: N83.6  Hematoma of broad ligament: N83.7  Hematometra: N85.7  Excessive bleeding in the premenopausal period: N92.4 |
| **Intraocular bleeding** | Retinal hemorrhage: H35.60-H35.63  Vitreous hemorrhage: H43.10-H43.13  Unspecified choroidal hemorrhage: H31.301- H31.303, H31.309  Expulsive choroidal hemorrhage: H31.311- H31.313, H31.319 |
| **Hemopericardium** | I31.2 |
| **Hemarthrosis** | M25.00-M25.08 |
| **Procedures** | **ICD-10 PCS code** |
| Percutaneous coronary intervention | 0270, 0271, 0272, 0273, 02C0, 02C1, 02C2, 02C3, 02H0, 02H1, 02H2, 02H3, 02N0, 02N1, 02N2, 02N3, 02Q0, 02Q1, 02Q2, 02Q3, 02U0, 02U1, 02U2, 02U3 |
| Coronary angiography | B200, B201, B202, B203, B207, B208, B20F, B210, B211, B212, B213, B217, B218, B21F |
| Right heart catheterization | 4A0239Z, 4A023N6, 4A023N8, 4A028N6, 4A028N8, 4A0289Z, 4A1239Z, 4A1279Z, 4A1289Z |
| CABG | 0210, 0211, 0212, 0213 |

Abbreviations: ICD-10 CM: International Classification of Diseases, Tenth Revision, Clinical Modification, ICD-10 PCS: International Classification of Diseases, Tenth Revision, Procedure, STEMI: ST-segment elevation MI, NSTEMI: non-ST-segment elevation MI, CABG: coronary artery bypass grafting

**Supplemental Table 2:** Utilization of mechanical ventilation, invasive testing, MCS, and revascularization in CS due to AMI amongst those with T1DM, T2DM, and no DM.

|  | **CS without DM**  **(N=204,303)** | **CS with T1DM**  **(N=4,289)** | **CS with T2DM**  **(N=149,809)** | **P-value** |
| --- | --- | --- | --- | --- |
| **Mechanical ventilation** | 96,856 (47.4%) | 2,075 (48.4%) | 73,289 (48.9%) | <0.001 |
| **RHC** | 34,234 (16.8%) | 859 (20.0%) | 27,455 (18.3%) | <0.001 |
| **LHC** | 119,486 (58.5%) | 2,660 (62.0%) | 90,441 (50.4%) | <0.001 |
| **Any MCS** | 73,735 (36.1%) | 1,604 (37.4%) | 55,854 (37.3%) | <0.001 |
| IABP | 52,384 (25.6%) | 1,179 (27.5%) | 40,646 (27.1%) | <0.001 |
| Impella | 23,307 (11.4%) | 499 (11.6%) | 17,806 (11.9%) | 0.012 |
| ECMO | 6,129 (3.0%) | 125 (2.9%) | 3,374 (2.3%) | <0.001 |
| **Any revascularization** | 108,152 (52.9%) | 2,248 (52.4%) | 79,901 (53.3%) | 0.300 |
| PCI | 82,042 (40.2%) | 1,454 (33.9%) | 54,284 (36.2%) | <0.001 |
| CABG | 28,573 (14.0%) | 836 (19.5%) | 27,508 (18.4%) | <0.001 |

Data are presented as count (percentage) and compared (chi-squared test)

Abbreviations: RHC = right heart catheterization, LHC = left heart catheterization, MCS = mechanical circulatory support, IABP = intra-aortic balloon pump, ECMO = extracorporeal membrane oxygenation, PCI = percutaneous coronary intervention, CABG = coronary artery bypass grafting

**Supplemental Table 3:** Adjusted odds of in-hospital outcomes among patients with AMI CS (reference group is CS without DM)

|  | **Type 1 DM** | **Type 2 DM** |
| --- | --- | --- |
| **In-hospital mortality** | 1.04 (0.94 – 1.15) | 1.133 (1.11 – 1.16) |
| **Urgent 30-day readmission** | 1.52 (1.33 – 1.74) | 1.262 (1.22 – 1.31) |

Data are presented as OR (95% CI). Data do not account for competing risks of outcomes
